# Integrated *Aedes* management for the control of *Aedes*-borne diseases

**DOI:** 10.1371/journal.pntd.0006845

**Published:** 2018-12-06

**Authors:** David Roiz, Anne L. Wilson, Thomas W. Scott, Dina M. Fonseca, Frédéric Jourdain, Pie Müller, Raman Velayudhan, Vincent Corbel

**Affiliations:** 1 MIVEGEC, IRD, CNRS, University of Montpellier, Montpellier, France; 2 Department of Biosciences, Durham University, Durham, United Kingdom; 3 Department of Entomology & Nematology, University of California, Davis, California, United States of America; 4 Center for Vector Biology, Rutgers University, New Brunswick, New Jersey, United States of America; 5 Department of Epidemiology and Public Health, Swiss Tropical and Public Health Institute, Basel, Switzerland; 6 University of Basel, Basel, Switzerland; 7 Department of Control of Neglected Tropical Diseases (HTM/NTD), World Health Organization (WHO), Geneva, Switzerland; University of Heidelberg, GERMANY

## Abstract

**Background:**

Diseases caused by *Aedes*-borne viruses, such as dengue, Zika, chikungunya, and yellow fever, are emerging and reemerging globally. The causes are multifactorial and include global trade, international travel, urbanisation, water storage practices, lack of resources for intervention, and an inadequate evidence base for the public health impact of *Aedes* control tools. National authorities need comprehensive evidence-based guidance on how and when to implement *Aedes* control measures tailored to local entomological and epidemiological conditions.

**Methods and findings:**

This review is one of a series being conducted by the Worldwide Insecticide resistance Network (WIN). It describes a framework for implementing Integrated *Aedes* Management (IAM) to improve control of diseases caused by *Aedes*-borne viruses based on available evidence. IAM consists of a portfolio of operational actions and priorities for the control of *Aedes-*borne viruses that are tailored to different epidemiological and entomological risk scenarios. The framework has 4 activity pillars: (i) integrated vector and disease surveillance, (ii) vector control, (iii) community mobilisation, and (iv) intra- and intersectoral collaboration as well as 4 supporting activities: (i) capacity building, (ii) research, (iii) advocacy, and (iv) policies and laws.

**Conclusions:**

IAM supports implementation of the World Health Organisation Global Vector Control Response (WHO GVCR) and provides a comprehensive framework for health authorities to devise and deliver sustainable, effective, integrated, community-based, locally adapted vector control strategies in order to reduce the burden of *Aedes*-transmitted arboviruses. The success of IAM requires strong commitment and leadership from governments to maintain proactive disease prevention programs and preparedness for rapid responses to outbreaks.

## Author summary

*Aedes aegypti* and *A*. *albopictus* are mosquito species that thrive in towns and cities and can transmit viruses to humans that cause diseases, such as dengue, Zika, chikungunya, and yellow fever. The geographic range of human infection with these viruses is rapidly expanding globally. Even when preventative or therapeutic treatments are available to fight these diseases, controlling the mosquito vector will remain an important control option. We therefore developed a framework called IAM that offers decision-making guidance based on available evidence of effective control of *Aedes* at different levels of infestation and virus transmission risk. Our work aims to strengthen the capacity of countries at risk of and/or affected by these diseases and vectors so they will be better prepared for existing and emerging *Aedes*-borne disease threats.

## Introduction

During the past 50 years, *Aedes*-borne diseases, such as dengue, Zika, chikungunya, and yellow fever, have emerged and/or reemerged globally [1]. Dengue virus is on the rise, causing about 390 million human infections per year; chikungunya virus spread worldwide in the early 2000s; Zika virus spread worldwide in the past three years; and yellow fever has resurged in Africa and the Americas [1, 2]. The expansion of these diseases can be explained in part by an intensification of the conditions favouring the dispersal and proliferation of *Aedes* as a result of global trade and unplanned urbanisation; inefficient implementation of vector control programmes due to inadequate human, financial, and infrastructural capacities; erratic water supply and associated water storage practices; ineffective waste disposal; and a lack of community engagement and political will [3, 4, 5]. All of the viruses that cause these diseases are transmitted primarily by the tropical yellow fever mosquito *Aedes aegypti*, and to a lesser extent by *A*. *albopictus*, the Asian tiger mosquito, of which there are both temperate and tropical strains [2]. The total global economic impact of *Aedes* vectors and related diseases is still unknown [6], but economic losses due to dengue have been estimated to be at least US$ 9 billion annually [7].

Although there is a vaccine for yellow fever, there are currently no commercially available drugs or vaccines for Zika or chikungunya. A dengue vaccine (Dengvaxia), developed by Sanofi-Pasteur, has been approved in several countries but has safety concerns for mass administration [8]. Moreover, new viruses may potentially emerge that could be transmitted by these vectors. Preventing or reducing disease caused by currently recognised or novel *Aedes*-borne viruses on a global scale continues to depend largely on controlling mosquito vector populations or interrupting human–vector contact.

Historically, well-implemented vertical *Aedes* control programmes were successful in controlling yellow fever in the Americas (1900s to 1960s) and, more recently, dengue in Singapore (1970s to 1980s) and Cuba (1980s to 1990s) [9]. Unfortunately, the recent resurgence of *Aedes*-transmitted arbovirus outbreaks throughout the world highlights the limitations of vector control, as currently deployed, to reduce the incidence of disease [1, 4, 10].

Efforts to address this increasingly urgent challenge have been boosted by a renewed focus on strengthening vector control, as witnessed at the May 2017 World Health Assembly, where the Global Vector Control Response (GVCR) received strong support from member states [11]. The GVCR provides countries with high-level, strategic guidance to reduce the burden and threat of vector-borne diseases—including *Aedes*-borne viruses—through effective, locally optimised, sustainable vector control. It aims to strengthen 2 foundations of vector control: (i) basic and applied research, and (ii) capacity and skill development. Building on these foundations, the GVCR advocates 4 pillars of action to be undertaken by countries: (i) vector surveillance and monitoring and evaluation (M&E); (ii) integrated application of control tools and approaches; (iii) engagement and mobilisation of communities; and (iv) inter- and intrasectoral collaboration. A number of enabling factors are required to achieve the desired results, including strong country leadership for resource mobilisation.

Despite this fresh impetus, many countries are still unprepared to address the challenge of *Aedes*-borne diseases and lack practical guidance on how and when to deploy vector control interventions in different entomological and epidemiological settings. This review was conducted by members of the Worldwide Insecticide resistance Network (WIN) [10] with the aim to support implementation of the GVCR by offering detailed recommendations for: (i) integrated vector and disease surveillance, (ii) vector control strategies, (iii) social mobilisation, and (iv) multisectoral approaches, providing a framework targeted to *Aedes* distribution and level of disease risk. We offer evidence-based guidance to implementing Integrated *Aedes* Management (IAM) systems and for strengthening national capacities so that public health programmes are better prepared for the emerging threat of *Aedes*-borne diseases.

### Decision-making based on transmission risk and *Aedes* distribution scenarios

**Fig 1:**
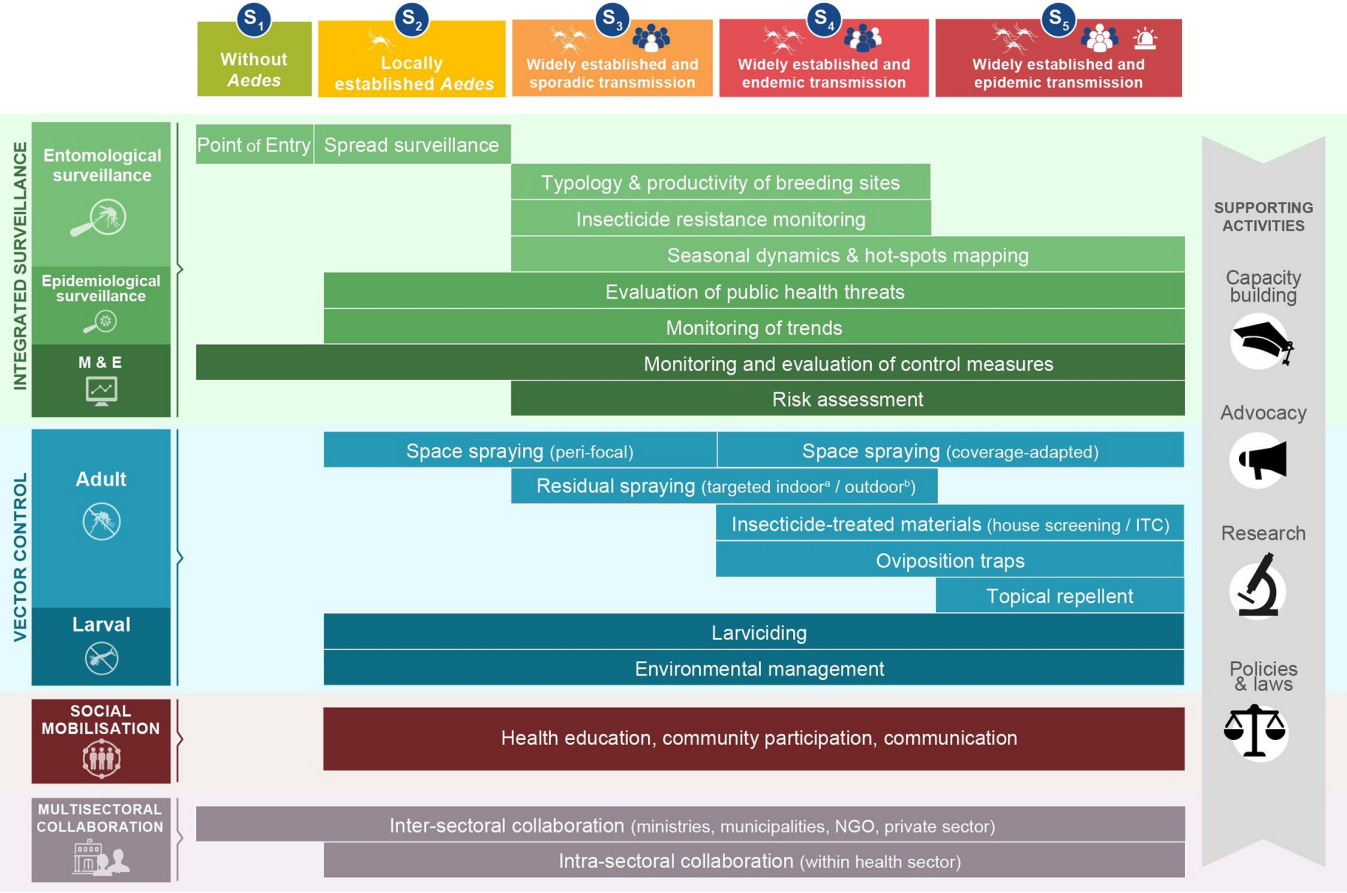
Conceptual framework of the IAM system. IAM builds on 4 pillars of activities (integrated surveillance, vector control, social mobilisation, and multisectoral collaboration) and 4 supporting activities (capacity building, advocacy, policies and laws, and research). Activities are tailored to local scenarios of *Aedes* distribution and virus transmission risk (see Box 1 for definition of terms). ^a^*A*. *aegypti;* ^b^*A*. *albopictus*. IAM, Integrated *Aedes* Management; ITC, insecticide treated curtain; M&E, monitoring and evaluation; NGO, nongovernmental organisation; S, scenario.

IAM proposes to tailor vector control responses according to the following 5 scenarios based on the local stage of *Aedes* distribution and level of virus transmission risk: Scenario 1 (or S_1_), no *Aedes* present (and no transmission); S_2_, *Aedes* locally established and no transmission; S_3_, *Aedes* widely established and sporadic transmission; S_4_, *Aedes* widely established and endemic transmission; and S_5_, *Aedes* widely established and epidemic transmission (Fig 1; Box 1). Risk scenarios are not fixed in space (at country, province, or district levels) or time and are likely to evolve based on updates in entomological and epidemiological risk assessment. The IAM aims to provide ‘graduated’ responses according to the risk level, but the switch from one scenario to another does not systematically follow a ‘linear transition’. For example, Key West, Florida, transitioned directly from S_3_ to S_5_ during the 2017’s Zika outbreak. Similarly, La Reunion Island and Italy switched from S_3_ to S_5_ during the chikungunya outbreak in 2007. Note that S_4_ (‘endemic transmission’) will typically be applied to viruses that have been established in a given location of some time, e.g., endemic dengue. A novel introduction and spread of a new arbovirus (e.g., Zika or chikungunya) or a new dengue serotype can rapidly produce a transition to an outbreak (S_5_).

**Box 1. Definition of key terms**

- ***Aedes***: We use the term *Aedes* for those members of the *Aedes* subgenus Stegomyia, the principal arbovirus vector *A*. *aegypti* and the secondary vector *A*. *albopictus*, although our approach may be equally applicable to other container-inhabiting vectors, such as *A*. *polynesiensis*, *A*. *japonicus*, or even other species.
- **Locally established**: Indicates a situation in which the species has colonised an area of less than 25 km^2^ [52].
- **Widely established**: Indicates a situation in which the species has colonised an area of more than 25 km^2^. European guidelines recommend an area greater than 25 km^2^ for the ‘widely established’ surveillance of invasive mosquitoes [52].
- **Sporadic transmission**: Refers to a situation in which autochthonous transmission occurs locally, irregularly, and unpredictably through viraemic travellers returning from disease-endemic countries [21, 53].
- **Endemic transmission**: Refers to the constant occurrence of cases of *Aedes*-borne disease limited in space but not in time (sustained temporal transmission) and where the highest number of cases is reported during a particular season [53, 54].
- **Epidemic transmission**: Refers to the occurrence of human cases of *Aedes*-borne viruses limited in space and time and clearly in excess of the number of cases normally expected [21, 53].

### Integrated surveillance and M&E

Integrated surveillance is an ongoing systematic collection, recording, analysis, interpretation, and dissemination of data to aid control efforts for initiating suitable public health interventions for prevention and control, including the M&E of the implemented control measures [12]. Collecting and using data in this way is intended to support assessment of risk for introduction and spread of vectors and viruses and to monitor and evaluate the control efforts followed by adjustments over time if necessary in accordance with predefined indicators (Table 1 and S1 Table). Entomological and epidemiological surveillance data should be promptly integrated, which will require efficient collaboration between vector-control and public health programs, and made available on a shared, easily accessible platform (e.g., the DHIS2, https://www.dhis2.org/overview).

**Table 1:** Integrated surveillance and M&E of *Aedes* and *Aedes*-borne diseases.

Tasks	Objectives		Description	Methodology/specifications	References
**Entomological surveillance**	Surveillance of routes of entry and monitoring the spread		The main routes of *Aedes* introduction and spread are harbours, international airports, accumulation in imported goods (such as tyres or lucky bamboo plants), borders, ground crossings, and traffic corridors (such as highways).	Ovitraps, oviposition traps (AGOs and GATs) and BG-Sentinel traps are recommended for points and/or routes of entry. These can be complemented by larval collection at relevant sites. Active monitoring of the spread is implemented with a network of oviposition traps. Passive surveillance is based on citizens’ reports. Mobile and web applications recommended for data entry and recording.	[52, 55]
	Seasonal dynamics and mapping (hot-spot identification)		Identification of suitable periods and areas of mosquito activity and abundance.	Weekly, biweekly, or monthly sampling all year round and in the same sites preferably with adult traps (BG-Sentinel) or ovitraps and in particular cases other methods (sticky traps, GAT traps, aspirator). Data can be related to climatic, landscape, demographic, and epidemiological variables. This task can be used to measure seasonal variations on vector density and better address relationships between entomological and epidemiological outcomes. GIS and mobile apps can be useful for mapping spatial heterogeneity of mosquito populations. Mathematical models can be used to predict potential expansion of vectors and location of hot-spots.	[14, 16, 22, 34]
	Typology and productivity of habitats suitable for mosquito larval development (breeding sites)		Identification of main larval development habitats and most productive containers to guide larval control actions.	Larval surveys are useful to investigate the presence and abundance of immature stages and to characterise the typology of larval development habitats. Pupal surveys are relevant for measuring productivity indices. This helps to identify the most productive containers for targeting larval control measures.	[13, 16, 56]
	Insecticide resistance monitoring		Monitoring the susceptibility of *Aedes* vector populations to public health insecticides.	WHO cylinder tests and CDC bottle assays are recommended for monitoring insecticide resistance at adult stages. WHO larval bioassays are recommended for immature stages. Resistance phenotype assays (frequency of resistance) and intensity bioassays (levels) are both recommended.	[18, 26, 49, 52]
**Epidemiological surveillance**	Evaluation of public health threats		Passive and active case detection depending on human and financial resources.	In addition to national routine surveillance system(s), enhancement strategies such as sentinel networks (hospitals, physicians, clinics, etc.) should be established for rapid laboratory diagnosis (RDT, molecular and serological tests, etc.) and to give notification of cases to vector control programs. Laboratory-based surveillance can contribute to distinguishing between imported and autochthonous cases. Laboratories with strong diagnostic capacity are needed to rapidly identify strains and/or serotypes and assess the potential for outbreaks.	[12, 13, 20, 57, 58]
	Guiding public health actions		Epidemiological data (incidence, morbidity, mortality) are used to prioritise and target public health actions, such as vector control, communication, and vaccination. In the case of an epidemic alert, passive surveillance can be enhanced to active surveillance with strengthen case detection.	Epidemiological data are obtained from surveillance, as described above. Active case detection (through door-to-door surveys or questioning by public health professionals) can be used to identify infected people who have not sought treatment, i.e., undiagnosed and under-reported cases. Monitoring the movements of population (i.e., seasonal migration) between different environments (from forest or rural to urban areas or cities) can be crucial to respond to virus introduction.	[12, 13, 19, 20, 21, 27, 58]
	Monitoring trends in public health burden		The health burden can be assessed at different levels (from local to national to regional) by recording clinical manifestations and by sorting cases according to relevant criteria, e.g., sex and age group. Results can facilitate the assessment of the disease and associated risk factors.	Although active surveillance provides more accurate data, monitoring can be used to assess health impact. Mandatory reporting of high quality data is crucial to support collection of information on demographics and transmission risks.	[13, 27, 58]
**Risk assessment**		Risk assessment based on adequate entomological and epidemiological surveillance is key to identifying areas of potential risk of *Aedes*-transmitted virus outbreaks and/or *Aedes* introduction and spread. Risk assessment will be used to define a preparedness and response programme tailored to the local situation and to monitor and evaluate the vector control programme. It is also useful for raising the different stakeholders’ awareness of the plausibility and extent of the expected risks. If the situation changes, the risk assessment should be reexamined.		Five steps are required: (1) identification of hazard(s), (2) likelihood of the virus and/or vector being introduced into a specific location, (3) spread or probability of transmission in the at-risk area, (4) probability the virus and/or vector will persist over a prolonged period, and (5) effects on health and the economy.	[52]
**M&E of control measures**		Monitoring refers to continuous tracking of programme performance. It makes it possible to find out whether activities have been implemented as planned, ensures accountability, and brings to light any problems or constraints so that corrective action can be instigated. Evaluation refers to the periodic measurement of the programme’s outcomes (e.g., vector populations) and impacts (e.g., reduced infection or disease) using data from the entomological and epidemiological surveillance systems.		Programmes should set up a logical framework that include predetermined indicators with defined data sources to measure programme performance through M&E. Data sources are typically entomological and epidemiological surveillance systems as well as programme reports and other records on delivery of interventions and behaviour change activities. These data should be available to entomology and public health programmes on a shared, integrated, easily accessible platform.	[13, 25, 29, 30, 31, 41, 48, 58]

**Abbreviations:** AGO, autocidal gravid oviposition traps; app, application; BG-Sentinel, BioGents sentinel traps; CDC, Centers for Disease Control and Prevention; GAT, gravid *Aedes traps*; GIS,Geographic Information System; M&E, monitoring and evaluating; RDT, Rapid Diagnostic Tests; WHO, World Health Organisation.

### Entomological surveillance

Surveillance of *Aedes* vectors is important for identifying changes in geographic distribution, to obtain relative measurements of variation in vector density over time, to facilitate appropriate and timely decisions regarding interventions, and to assess the entomological impact of mosquito control programs to see whether the intervention had the expected effect on the target mosquito population [13]. For routine surveillance, entomological measurements have to be done in the same location (sentinel sites) at regular time intervals in order to establish a baseline to follow variation over time (seasonal dynamics). The frequency of data collection should be based on programme capacity and the need to generate reliable data in an appropriate format. Given that the geographic distribution of *Aedes* is increasing globally, a systematic surveillance for *Aedes* in every country is needed. Surveillance at points and/or routes of entry, such as sea ports, airports, and land country borders is important for early detection of the introduction of invasive *Aedes* species (S_1_, Table 1). If mosquitoes are introduced into suitable habitats, they may become established locally (S_2_) or more widely (S_3_, S_4_, S_5_). At this stage, surveillance consists of monitoring the spread of the mosquitoes (e.g., using ovitrap networks) in order to identify areas and/or periods of high transmission risk based on vector infestation, e.g., mapping seasonal dynamics and disease hot-spot identification [14]. The presence and abundance of *Aedes* species are estimated from measures of different entomological indices (e.g., larvae, pupae, adult), each with their strengths and weaknesses, as summarised in S2 Table. It should be mentioned, however, that cross-sectional studies have failed to find good correlations between entomological indices and episodes of dengue [15], and no larval entomological thresholds have proven effective in predicting *Aedes*-borne virus epidemics [16]. This can be explained by the fact that dengue virus transmission is complex and varies through time and space, and the relationship between vector density and risk of human infection is not static nor adequately characterised through periodic entomological surveillance [15].

New technologies (i.e., geo-informatics tools, remote sensing, and mathematical and simulation models) can be helpful in mapping the spatial distribution of vectors and/or in predicting their spread and seasonal dynamics using climatic (e.g., temperature, rainfall), social (e.g., rent value or education level), demographic (e.g., population density or distance to urban habitats), and landscape (e.g., vegetation cover or type of urbanisation) variables and can be less expensive than field surveillance [17]. A good understanding of the typology and productivity of habitats suitable for mosquito larval development (S_3_, S_4_) is essential in order to target larval control operations. Due to the possibility of introduction or selection of resistant individuals, insecticide resistance should be monitored regularly, preferably during nonepidemic periods (S_3_ and S_4_) to guide the choice of insecticides used for mosquito control. A combination of biological, biochemical, and molecular tools can be used to measure the frequency, intensity, and mechanisms of insecticide resistance in natural populations. Each resistance testing tool has its own advantages and weakness [18].

In areas with virus transmission (S_3_, S_4_, S_5_), if resources permit, it can be helpful to screen vectors for virus infection in order to confirm the role played by suspected species in local transmission, to monitor spatial and temporal patterns in virus transmission dynamics, and to evaluate interventions. Because virus detection in mosquitoes is costly and time-consuming and finding infected mosquitoes in natural populations is often challenging, it is not regularly done for surveillance of *Aedes*-transmitted viruses [19].

### Epidemiological surveillance

In the field of *Aedes*-borne viral diseases, the objectives of epidemiological (or human and possibly animal) surveillance are to (1) evaluate potential public health threats, carrying out risk assessments and detecting outbreaks early; (2) select and evaluate the effectiveness of control activities; and (3) monitor trends in public health burden to obtain data for assessing the social and economic impact on the affected community (Table 1) [20].

The threat to public health is assessed by identifying recent introductions of a virus (S_3_), monitoring travellers returning from areas where target viruses circulate (S_3_, S_5_), and mapping local spread of the virus (S_4_, S_5_). This kind of surveillance system requires robust indicators and action plans to be defined in order to stratify epidemiological risk and guide decision-making to facilitate switching from one epidemic scenario to another [21].

Epidemiological data play a key role for guiding and prioritising vector control responses, which will be graduated according to transmission risk (Fig 1). Firstly, attention should be focussed on timely detection of imported viraemic people (S_3_), followed by identifying hot-spots of virus transmission (S_4_, S_5_). Monitoring geographical and temporal trends in human cases is common to all scenarios. The main differences lie in the targets of surveillance—trends in virus importation (mainly for S_3_ and S_5_), trends in circulating strains or serotypes, disease incidence, morbidity and mortality (S_4_ and S_5_). Evidence of non−vector-borne transmission (e.g., sexual transmission and blood transfusions) should be investigated where appropriate (e.g., Zika infections) as well as potential sylvatic transmission of the viruses, e.g., yellow fever. In geographical areas where local virus circulation is already established (S_4_, S_5_), a significant proportion of all suspected cases should be confirmed by specific laboratory diagnostics, depending on local resources [13].

Epidemiological surveillance is most often based on a combination of active and passive surveillance in order to reconcile cost, sensitivity, response time, and geographical coverage. Depending on the purpose of the epidemiological surveillance system and the risk scenario, certain considerations—such as available resources (e.g., human and diagnostic capacities) or strength of the healthcare system (e.g., public and/or private and accessibility), and sensitivity or response capacity—will be critical for guiding stakeholders in their choice of design of the overall disease management system (Table 1).

Epidemiological data, however, have several limitations. The most notable challenges are a high proportion of people with asymptomatic and/or mild infections, or differential diagnosis with low specificity of symptoms; a broad range of disease manifestations, from no detectable illness to death; lack of standardisation in case definitions; limited or low diagnostic capacity; underreporting; and variation in treatment-seeking behaviour by infected people [22]. Active case detection in the surroundings of a person with a confirmed infection may help identify additional cases or clusters, which often go unreported or undiagnosed. Where there is an epidemic alert, passive surveillance can be enhanced to reduce delays in reporting cases or to extend the area of surveillance. In areas at risk of sporadic transmission (S_3_), healthcare personnel usually have to report cases of imported and autochthonous arboviral disease, such as dengue, chikungunya, or Zika, to public health authorities. Increasing awareness among clinicians and travellers returning from endemic areas combined with good laboratory capacity has greatly improved case reporting. Laboratory-based surveillance has been shown to play a role in monitoring the introduction of a novel dengue virus serotype, a switch of virus strains between vector species and cocirculation of different arboviruses [22]. In nonendemic and/or nonepidemic areas (S_2_, S_3_), surveillance can target imported cases because these represent the main threat for introduction into immunologically naive populations. This can be achieved by health professionals notifying the relevant authorities of suspected or confirmed imported cases [23] or by fever screening travellers at points of entry [24].

### Risk assessment and M&E outcomes

Evidence-based risk assessment should be carried out within all risk scenarios (S_1_−S_5_) and should be conducted by national and international health (and environmental) agencies. The assessment should form the basis for developing guidelines for the actions needed to keep risk to a minimum. To our knowledge, there is no global framework for conducting risk assessment for *Aedes*-borne diseases, but several regional documents have been drawn up (see Table 1). Regular M&E of the delivery of dengue prevention and control services and of the impact of interventions (this one being a critical one) are important IAM activities in all scenarios. Suitable indicators for measuring the progress of implementation (e.g., intervention coverage) and the outputs and outcomes (e.g., reductions in vector density or disease) should be identified [13].

### Vector control

Vector control efforts need to be sustained over time, which requires well-structured administration, coordination with the public health programme that is diagnosing cases, political will, skilled staff, funding, and, crucially, community engagement and mobilisation from the outset [4]. Vector control can be undertaken either as a ‘routine’ activity (i.e., a preemptive sequence of actions regularly carried out) or as an ‘emergency’ measure (i.e., a response to an excess of vectors and/or an unusual increase in the human disease incidence calling for immediate action). Both types of measures should be prepared for, but vector control is most cost-effective if it is ‘proactive’ (preventive) rather than implemented ‘in response mode’ (after the start of epidemic) [25]. Because programmes move from areas prone to virus introduction (S_3_) to endemic-epidemic (S_4_−S_5_) scenarios, a shift in the allocation of resources from ‘reactive’ to ‘proactive’ vector control should be considered. Targeting immature mosquitoes has been a prevalent paradigm for *Aedes* control, but far more attention should be directed at methods targeting both larvae and adults to maximise impact on adult *Aedes* density, longevity, and role in virus transmission [9]. Strategies and interventions should be adapted to local vector ecology and available resources, guided by results from operational research and subject to routine M&E (see the ‘Risk assessment and M&E outcomes’ section).

In order to prolong the life span of existing insecticides, it is imperative that noninsecticide-based tools are used whenever possible. When chemicals have to be deployed, they should be used rationally and preferably not as ‘monotherapies’ [10, 26]. Several insecticide resistance monitoring strategies exist in vector control, which are based on rotations of insecticides, mixtures of unrelated insecticides, use of interventions in combination, and mosaic spraying. Resistance management is not a ‘stand-alone’ strategy and should be implemented in the broad context of integrated vector management and be carefully monitored and evaluated. Activities to control transmission should target homes and outdoor areas in their immediate vicinity (i.e., in the place of residence as well as in neighbouring houses). Treating nonresidential areas—i.e., places where human–vector contact occurs during the daytime, such as schools, hospitals and workplaces, especially their surroundings, such as outside lunch gathering areas—can provide measurable impacts [27]. Restricting control to residences within a certain radius of a case’s home is not as effective as uniform treatment of broad geographic areas. By the time a case is detected, human movement has taken the virus beyond a radius of 100 m to 200 m [27, 28].

### Evidence supporting *Aedes* vector control

The evidence base for the public health value of *Aedes* vector control is unfortunately weak. There are little data demonstrating reduction in human infection or disease for many tools currently in widespread use [29]. Epidemiological outcomes are needed to demonstrate the public health benefit of a vector control intervention and are the basis of evidence-based policy [30]. In order to provide more robust guidance on preferred *Aedes* vector control tools and those that should be avoided, we summarised existing evidence based on recent systematic reviews. We categorised the hierarchy of evidence according to whether there was epidemiological or entomological evidence and by study design, with randomised controlled trials providing the highest quality of evidence and nonrandomised or observational studies providing the lowest quality evidence [30]. Results and specified details for different interventions are shown in S2 Table.

### Adult mosquito control and avoidance

The strengths and limitations of current adult mosquito control methods and the strength of evidence for their entomological and epidemiological effects are summarised in Table 2. Despite widespread use, there is limited entomological and epidemiological evidence for ultra-low volume (ULV) space spraying [31, 32]. In the case of virus transmission (S_3_, S_4_, S_5_), ‘peridomestic’ or ‘perifocal’ space spray treatments with insecticides can be carried out in and around households where human infection is suspected or has been reported. Space sprays can also be adequate in specific situations, for example, (i) to prevent local establishment of invasive mosquito species, such as *A*. *albopictus* (small area < 25 km^2^, S_2_), (ii) to halt an incipient outbreak (S_3_), and (iii) to curtail an ongoing epidemic and/or endemic situation (S_4_ and S_5_). Different treatment methods (house-to-house application using portable equipment, vehicle-mounted fogging, and cold or thermal fogging) are available, but they must be tailored to the risk scenario, the area to be covered, accessibility, and the *Aedes* species.

**Table 2:** Vector control tools for *Aedes* mosquito control. Strength of evidence is based only on recent systematic reviews and meta-analysis studies carried out in the last 5 years. We used scores to rank the ‘strength of evidence’ based on study designs used for assessing the efficacy of vector control interventions as proposed by Wilson and colleagues (2015) for epidemiological trials (1, 2a, 2b). We created 2 new ‘levels of evidence’ (3a and 3b) to distinguish randomised versus nonrandomised (observational) ‘entomology’ trials.

Stage/scenario	Methodology	Type of intervention/product	Strength of evidence*	Constraints/advantages	Specifications	References
**Adult control in emergency S**_**2**_**, S**_**3**_**, S**_**4**_**, and S**_**5**_	**Insecticide spraying**	Space spraying (indoors, outdoors)	Epidemiological evidence for ISS based on observational studies (level 2b). Several entomological studies (level 3a and 3b) for ISS and OSS.	Insecticide resistance Low acceptability and limited sense of security in the community Poor persistence Regulatory and environmental constraints Needs skilled, experienced staff	Thermal fogging or cold fogging (ULV spray) using WHO-recommended insecticides Indoor house-to-house application using portable sprayer. Outdoor applications (i.e., vehicle-mounted fogger) if mosquitoes are exophilic and exophagic. Applications should be carried out at the right time, in the right place and according to prescribed instructions.	[13, 29, 31, 32, 34, 48, 58, 59]
		Residual spraying (indoors or outdoors)	Epidemiological evidence of IRS (level 2a). Entomological evidence (level 3b) for IRS for *A*. *aegypti* and ORS for *A*. *albopictus* (level 3b).	Insecticide resistance Costly and time-consuming Requires high coverage Needs skilled, experienced staff	TIRS for indoor resting *A*. *aegypti* ORS on the vegetation against *A*. *albopictus* Application by portable compression sprayers	[29, 31, 34, 35, 59, 60, 61]
**Adult control for routine and emergency S**_**4**_ **and S**_**5**_	**Mass trapping**	Gravid traps (AGO or GAT)	Epidemiological evidence based on observational studies (level 2b). Entomological evidence (level 3b) for *A*. *aegypti*.	Low cost Possible to combine with community participation Sustainable, able to be reused for several seasons	Need for a coverage of greater than 80% Use large autocidal gravid traps, as AGO or GAT, to maximise visual and olfactory attraction using grass or hay infusion	[36, 62, 63]
**Adult control for routine and emergency S**_**4**_ **and S**_**5**_	**Personal protection**	Topical repellents (applied directly onto the skin)	Absence of epidemiological and entomological evidence as a part of control campaigns.	Individual-based action (requires high degree of compliance) No residual activity	DEET, the longest-lasting; IR3535 or picaridin, medium-long lasting protection; plant-derived oils (eucalyptus, citronella, or geranium), short-term (frequency of applications according to national legislation and/or manufacturer’s recommendations)	[29, 64]
		Insecticide-treated materials (clothes, curtains, house screens, water container covers, etc.)	Epidemiological evidence for house screening (level 2b). Entomological evidence for ITCs, house screening, and water container covers (level 3a and 3b). No evidence for bed nets.	Individual- and community-based action Residual activity with long-lasting technology Insecticide resistance Low protection against UV Degradation of insecticide	Clothes, curtains, and bed nets treated with WHO-recommended insecticides. Most evidence supports house screening for preventing dengue transmission	[29, 65, 66]
**Larval control for routine S**_**2**_**, S**_**3**_**, S**_**4**_**, and S**_**5**_	**Environmental management**	Source reduction and educational outreach visits (door-to-door)	Epidemiological evidence (level 1) of community based campaigns. Entomological evidence (level 3a and 3b).	Labour intensive. Larval development habitats need to be accurately identified. Must be done diligently and conscientiously and with access to a high number of dwellings	Requires a high level of education and community participation. Difficult to sustain over time. Need to characterise larval development habitats, including urban cryptic habitats. Essential to reduce mosquito larval development habitats in the long-term in private and public domains	[29, 40, 41, 43, 48, 58, 67]
	**Larviciding**	Organophosphates (Temephos, Chlorpyrifos, Pirimephos methyl, Fenthion)	Entomological evidence for Temephos (level 3b).	Affordable Not acceptable for treating drinking water containers and sources (except Temephos) Temephos resistance in several areas Regulatory constraints (e.g., OPs are not notified in the EU for mosquito control)	Cholinesterase inhibitors Different formulations (EC, GR) and application methods (manual or with hand sprayers)	[41, 68]
		Insect growth regulators (pyryproxifen, diflubenzuron, novaluron)	Epidemiological evidence for pyryproxifen as part of community base (level 2b). Entomological evidence (level 3b).	More expensive Late acting effect (pupae) on juvenoids Acceptable for treating drinking water sources and containers Constraints for the treatment of cryptic breeding sites	Disruption of endocrine system for juvenoids (pyriproxyfen) and chitin synthesis inhibitor for ecdysoids (novaluron and diflubenzuron) Different formulations (WG, GR, DT) and application methods (manual or with hand sprayers)	[29, 39]
		Bti	Entomological evidence (level 3a and 3b) for Bti.	No resistance Selective and safe Acceptable for treating drinking water sources and containers Low residual action in polluted habitats	Bacterial toxins targeting midgut epithelium cells Different formulations (WG, GR) and application methods (manual or with hand sprayers and fogging).	[34, 69]
	**Biological control**	Fish (*Gambusia*, etc.)	Limited entomological evidence (level 3b) for fish.	Well accepted in several countries, needs a delivery mechanism and maintenance. Adequate for treating large and/or permanent mosquito habitats, not generally accepted for drinking water storage containers.	Predators of mosquito larvae (kill all stages). Controversial, harmful impacts of nonnative species, such as *Gambusia*.	[34, 41, 70, 71, 72, 73, 74]
		Copepods (*Mesocyclops*)	Limited epidemiological (level 2b) and entomological evidence (level 3b) for copepods depending on settings.		Predators of mosquito larvae (kill young instar larvae).	

*Details are available in S2 Table.

**Abbreviations:** AGO, autocidal gravid oviposition traps; Bti, *Bacillus thuriengensis* serotype *israliensis*; DEET, N,N-diethyl-3-methylbenzamide; DT, Tablet; EC, Emulsifiable concentration; EU, European Union; GAT, gravid *Aedes* trap; GR, Granules; IRS, indoor residual spraying; IR3535, Ethyl butylacetylaminopropionate; ISS, indoor space sprays; ITC, insecticide-treated curtain; OP, Operational procedures; ORS, outdoor residual sprays; OSS, outdoor space spray; S, scenario; TIRS, targeted indoor residual spraying; ULV, ultra-low volume; UV, Ultraviolet; WG, Water dispersible granule; WHO, World Health Organisation.

Indoor space spray (ISS) should be distinguished from outdoor applications. Because *A*. *aegypti* tends to be endophilic and endophagic [33], only in cases in which *A*. *albopictus* (or *A*. *aegypti*) populations are primarily outdoors are outdoor applications likely to be effective. Outdoor space spraying (OSS) and outdoor residual spraying (ORS) on vegetation have been used for controlling the exophilic species *A*. *albopictus* [34], with some entomological evidence of efficacy (Table 2, S2 Table). The efficacy of ORS and its impact on the environment is still controversial, facing the same challenges as described above for ULV space spraying.

Indoor residual spraying (IRS), in particular targeted IRS (TIRS, in which IRS is performed on exposed low walls, under furniture and inside closets) has not been widely used for *Aedes* control so far, although it may be a promising tool for controlling *Aedes*-borne arboviral transmission (S_3_ and S_4_) in areas where the endophilic mosquito *A*. *aegypti* is responsible for transmission [35]. The costs, human resources, and logistics needed for suitable coverage with IRS may represent a challenge for their rapid and broad-scale deployment during outbreaks. The use of contact tracing technologies to deploy IRS and/or TIRS can be an option to overcome those limitations [35].

In S_4_ and/or S_5_, local health authorities can promote or subsidise the use of insecticide-treated materials (e.g., insecticide house screening and treated curtains) that have been proven effective (S2 Table) and, in emergency situations (S_5_), promote the use of topical repellents that provide protection against mosquito bites (Table 2, Fig 1).

Finally, epidemiological and entomological evidence exist with regards to the mass deployment of gravid oviposition traps to reduce *Aedes* mosquito density (S2 Table) that can be a low-cost, community-based, and sustainable participation complementary strategy (Table 2) [36].

Furthermore, there is currently considerable innovation in vector control for prevention of *Aedes*-borne viral disease [37]. Novel approaches—such as the sterile insect technique, *Wolbachia*, genetically modified mosquitoes, removal trapping, and spatial repellents—gather relevant entomological data, and many are engaged in well-designed field trials that will generate the epidemiologic data necessary to develop public health policy for their deployment.

### Larval control

Methods of larval control and their strengths, limitations, and evidence base are described in Table 2. The aim of targeting mosquitoes at immature life stages (i.e., larvae and pupae) is to reduce adult *Aedes* emergence and to reduce adult population densities. Control may be intensified during the early mosquito season but necessary year-round in tropical regions and requires high coverage because there may be sufficient temporary larval habitats to maintain high mosquito adult densities and virus transmission. Larval control (i.e., environmental management, source reduction, larviciding, or biological control [community-based and/or top-down approaches]) is more effective when it is consistent and routine (S_4_) rather than in a periodic emergency response (S_5_). Larval control needs to be sustained in order to reduce the size of the adult vector population and to keep the population density below certain, currently still undefined thresholds to minimise the risk of virus transmission.

Source reduction has been, and continues to be, a key component of dengue, Zika, and chikungunya control programmes [9, 38]. It should primarily target artificial containers in private and public spaces, although some natural containers, such as bamboo and bromeliads, can also harbour *Aedes* larvae. Larvicides are generally long-lasting and moderately costly. Unfortunately, the evidence supporting larviciding as part of control programmes is mixed, with some studies showing a beneficial effect of pyryproxifen as part of a community-based strategy reducing dengue rates and entomological indices [39], whereas others such as Temephos or *Bacillus thuriengensis* (Bti) do not have strong evidence in the review of evidence (S2 Table and Table 1).

Biological control methods (fish, copepods, and others) are relatively acceptable and can be used for treatment of large and permanent breeding sites, but existing evidence is inadequate for assessing the impact of this strategy for dengue control (S2 Table). Community-based source reduction (as clean-up campaigns or the use of water container covers) can reduce *Aedes* vector populations and is supported by epidemiological cluster randomised controlled trials results of lower infection with dengue virus in children and fewer reports of dengue illness from a trial in Mexico and Nicaragua (Table 2 and S2 Table) [40, 41].

### Social mobilisation

Human behaviour is the common denominator of all *Aedes*-borne virus epidemic risk scenarios and therefore of prevention and control strategies. Social mobilisation is a key factor in the success of *Aedes* mosquito control strategies and in preventing outbreaks. There is evidence (S2 Table) that community participation is effective in reducing larval indices and disease prevalence [40, 41, 42, 43]. Community participation and education (e.g., door-to-door visits, workshops, and webinars) can inform the population on how to reduce *Aedes* populations by emptying or eliminating nonpermanent water containers and covering permanent water storage containers with untreated or insecticide-treated covers. Other actions can be carried out by health education programmes, such as distribution of printed materials, educational meetings, involvement of local opinion leaders, sensitisation at schools, and the use of mass media (radio, television, newspapers, leaflets, posters) [42]. Health education efforts should be carried out routinely and intensified before peak periods of virus transmission (S_4_ and S_5_). Sex education is also important for Zika prevention because the virus can be transmitted sexually.

WHO recommends the use of communication for behavioural impact (COMBI), an approach that integrates behavioural and social communication to reduce risk and prevent disease. COMBI is used in an increasing number of countries for dengue control [44], and a toolkit has been developed to deliver more effective outbreak response measures [45]. In practice, education and communication strategies are often implemented too late, that is, after the outbreak has begun to decline (S_4_ and S_5_). Social communication is more likely to be successful when information is disseminated early, which means before the introduction of vectors or virus (S_1_), when transmission has recently been established (S_2_), or before transmission has peaked (S_1_, S_2_, S_3_ and S_4_). Activities to promote behavioural change should produce measurable and visible results and should be monitored using appropriate indicators [44]. It is important to note that social mobilisation is not a ‘stand-alone’ strategy and that community-based control campaigns are carried out in combination with other vector control interventions [29, 41, 42, 43].

### Intra- and intersectoral collaboration

*Aedes* control cannot be successful without effective and sustained intra- and intersectoral collaboration [4, 13, 46]. Within the health sector, *Aedes* control should not be the responsibility of a single department. Interagency collaboration is fundamental for a successful programme. The vector control unit should, therefore, establish strong links with other vector-borne disease programmes (e.g., malaria vector control), epidemiological surveillance, clinical diagnosis and management, vaccine delivery (when appropriate), maternal and child health (e.g., integrated management of childhood illnesses), health education, veterinary surveillance, and environmental health [46]. Intersectoral actions for vector control (Table 3) should be guided by site-specific knowledge of larval aquatic habitats, locations where risk of infection is highest (i.e., inside homes for endophilic and endophagic *A*. *aegypti* or outside homes for exophilic and exophagic *A*. *albopictus*), and current and historical hot-spots of reported illness. The specific actions that can be taken in collaboration with other sectoral actors will depend on the setting and feasibility.

**Table 3:** Framework for promoting intra- and intersectoral collaboration.

Ministry or organisation	Scenario	Activity	Rationale
Ministry of Public Works and municipal authorities	S_1_, S_2_, S_3_, and S_4_	Provision of reliable piped water to households. Provision of sewage connections. Solid waste management and disposal. Design and maintenance of street storm water drainage systems that do not harbour immature vectors.	Removal of potential *Aedes* mosquito larval development habitats can reduce adult numbers and virus transmission rates.
Ministry of Housing	S_3_ and S_4_	Develop and enforce housing and building codes mandating installation of screening, dependable water supply, waste management, and disposal and rainwater runoff control in new housing developments.	Reduce biting of humans by installing screens to prevent entry to houses and buildings. Reduce standing water to prevent immature development.
Ministry of Education	S_3_ and S_4_	Incorporate information on *Aedes-*borne diseases, vectors, transmission, and prevention into school curricula and teach hygienic behaviour.	Empower children with knowledge and skills to reduce mosquito populations and virus transmission.
	S_3_, S_4_, and S_5_	Participation of school children in larval surveys, source reduction, and larviciding.	
Ministry of Tourism	S_3_ and S_4_	Reduce aquatic habitats in and around hotels, community gathering places, markets, etc.	Reduce virus transmission.
	S_5_	Communicate rapidly to holiday accommodation providers and tourists if there is evidence of an outbreak.	Supports rapid implementation of vector control measures.
Ministry of Agriculture	S_3_ and S_4_	Encourage livestock farmers to empty, clean, and scrub animal drinking containers weekly.	Reduces aquatic habitats for *Aedes* mosquito development.
Department of Agriculture or Customs	S_1_	Involved in surveillance of invasive mosquito species at PoEs.	Supports early detection of *Aedes* species introductions.
NGOs	S_3_ and S_4_	Promote and implement environmental management, health communication on source reduction and improvement of housing.	Mobilising community action supports for more effective control.
	S_5_	Strengthen mobilisation of communities during outbreaks.	
NGOs and UN agencies	S_3_, S_4_, and S_5_	Deliver vector control interventions in humanitarian crises.	Prevent outbreaks and reduce impact on vulnerable populations
Private sector	S_3_ and S_4_	Develop new tools to prevent transmission, e.g., mosquito-proof water containers, door and window screens.	Stewardship, particularly in industrial and manufacturing sectors, can stimulate innovation in vector control and help reduce virus transmission.
		Recycle containers, e.g., plastic receptacles and tyres, to reduce aquatic habitats.	
		Involve architectural practices in design and building of mosquito-proof housing, schools, and workplaces.	
		Include private health facilities in epidemiological surveillance reporting systems.	
	S_2_, S_3_, and S_4_	Conduct health impact assessments on large-scale industrial projects and commercial agriculture.	
	S_2_, S_3_, and S_4_	Implement control measures in large-scale industrial projects and commercial agriculture.	
	S_4_ and S_5_	Increase access to subsidised personal protection measures.	
Academic and research institutions	S_1_−S_4_	Provide training and a career path for vector control specialists in the Ministry of Health, and other vector control personnel.	Support innovation and research expertise to improve and sustain surveillance and vector control.
	S_1_−S_4_	Share infrastructure, such as entomology laboratories, insectaries, and equipment, with the Ministry of Health.	Resource sharing reduces costs for the Ministry, supports access to specialised facilities, and fosters collaboration between academic and public health sectors.

**Abbreviations:** NGO, nongovernmental organisation; PoE, point of entry; S, scenario.

Outside the public health sector, collaborations should be forged with, for example, ministries of education, environment, water, and urban planning and housing [5], and with the private sector, nongovernmental organisations (NGOs), and town councils (Table 3). For example, provision of reliable piped water should be encouraged to prevent storage of water in containers in and around the home, which can harbour *Aedes*. Solid waste management should be improved to remove rubbish from the peridomestic environment, which can accumulate water and provide habitats for *Aedes* to lay their eggs. A pilot study of recycling of plastic materials in Merida, Mexico, was able to reduce entomological indices by incentivised recycling. Bonus points were given for large volumes of reusable materials in exchange for commodities and targeted at the most at-risk neighbourhoods [47]. The scheme was organised by the local government through the Health Services and in coordination with the Ministries of Social Development, Urban and Environmental Development, and Education. The Ministry of Housing and Infrastructure can develop and enforce housing and building codes, for example, to mandate installation of screened doors and windows on properties and rainwater runoff control for new housing developments as well as prohibit construction of open groundwater wells. The Ministries of Education and Health can work together to disseminate behaviour change communication on prevention of *Aedes* population and disease proliferation (see the ‘Vector control’ section). Information on prevention of *Aedes*-borne diseases should be integrated into school curricula for long-term sustainability. NGOs, including community groups, such as neighbourhood women’s, religious, environmental, and social action organisations, should be engaged. Local community groups can be involved in promoting and implementing environmental management as well as delivering behaviour change communication. NGOs can be influential in mobilising communities and encouraging acceptance of routine and outbreak vector control methods (S_5_).

The private sector can be engaged. For example, commercial companies can support recycling, e.g., disposal and recycling of discarded tyres. In Brazil, a partnership between the Ministry of the Environment and the National Association of the Tyre Industry encourages consumers to return used tyres to collection points at which point they are used as alternative fuel or recycled into flooring and other products [13]. Private health facilities can be incorporated into the epidemiological surveillance system. Academic and research institutions can cooperate with the Ministry of Health to train personnel and carry out surveillance through the sharing of facilities, i.e., entomology laboratories, insectaries, and human resources. Development projects and commercial agriculture can undertake assessments of the health impact of *Aedes*-borne diseases and implement mitigation strategies. NGOs, UN agencies, and bilateral or multilateral donors can be engaged to implement control measures to prevent virus transmission in conflict zones.

All these activities should be coordinated through an interministerial steering committee with broad representation that seeks regular input from nonministerial stakeholders, such as NGOs, research and educational establishments, community organisations, and the private sector [13]. The committee should have clearly defined terms of reference and meet regularly, not just during outbreaks. Working in an integrated fashion has the potential to increase efficiency and public health impact more than narrowly focused, uncoordinated actions from the health sector alone.

### Supporting activities

Additional important complementary activities for achieving effective vector control and *Aedes*-borne disease prevention include capacity building, advocacy, policies and laws, and research and innovation (Fig 1). Supporting activities are briefly summarised in S3 Table.

## Conclusion

During the past 50 years, the world has seen the emergence and dramatic spread of *Aedes*-transmitted arboviral diseases. Social, environmental, and demographic changes have facilitated the proliferation of existing transmission systems and the spread of viruses and vectors into new ecological settings [4, 46]. Notable deficiencies in the planning and implementation of vector control programmes were reported and include the following:

- Lack of commitment and leadership from governments to maintain preparedness and deliver rapid response against *Aedes*-borne diseases [46].
- Ineffective programmatic implementation due to the difficulties in eliciting sustainable community engagement and the challenges of applying large, top-down approaches [4].
- A weak evidence base for the public health effectiveness of *Aedes* vector control strategies due to the small number of robust trials that have been carried out and the difficulty in measuring the impact of interventions on human infection and disease [29, 30, 48].
- Insufficient funding, human resources, and limited capacity building in low-income countries stifles development of innovative control tools and strategies [3, 37].
- Absence of a global and coordinated plan to monitor and manage insecticide resistance in *Aedes* to guide decision-making for vector control [10, 49].
- Increasing aversion of citizens to strategies based solely on insecticides because of their potential impacts on the environment and inadequate application coverage [26, 31].

The call for a global response and preparedness for vector borne diseases (i.e., GVCR) is, therefore, timely [11], and implementing and sustaining integrated surveillance and locally adapted *Aedes* control measures should be a priority [50]. Unfortunately, there have been very few well-conducted epidemiological field trials of *Aedes* vector control (S2 Table), which means that prioritisation of control strategies is difficult. Limited epidemiological evidence supports the deployment of community-based source reduction, ISS, TIRS, and house screening and larviciding, but evidence is lacking for most other vector control tools (S2 Table, Table 2). Promising new interventions targeting adults—such as *Wolbachia* for population replacement and/or population suppression, genetically modified mosquitoes, sterile insect techniques, community-based mass trap deployment, spatial repellents, and attractive targeted sugar baits—are currently being considered for dengue prevention, but their public health efficiency in field trials (i.e., effectiveness trials) has yet to be determined, and large-scale roll-out (effectiveness studies) of these programmes will take years [37, 51].

There are no magic bullets for *Aedes-*borne diseases control, including vaccines. The desire to find easy or rapid solutions has been tried for decades without success and is likely to continue to lead to disappointment. The most practical and productive path forward is to intensify surveillance and to strengthen the evidence base so that, when needed, ‘a box’ of effective tools can be deployed in an integrated manner that takes into account the local situation and available resources.

The IAM provides a technical guidance on how and when to implement integrated management for *Aedes*, tailored to location-specific entomological and epidemiological risk scenarios based on current evidence. To be effective and sustainable, IAM must be fed with robust entomological and epidemiological data collection at different time and spatial scales (country, district, city, and neighbourhood) and in particular for local scenarios in endemic and epidemic settings with randomised controlled trials of combinations of several tools, to guide-decision making for *Aedes*-borne disease control. The IAM needs be supported by human and financial resources and must be carefully monitored and evaluated. Increased funding is crucial given the growing threat to public health and the need for evidence that innovation makes disease prevention more effective. We hope that the measures outlined here will help promote and implement the WHO GVCR and will be used to guide actions that improve *Aedes*-borne disease prevention and outbreak response. The ultimate goal is to use available resources as effectively and efficiently as possible to safeguard global health.

**Key Learning Points**

- *Aedes*-borne viral diseases are rapidly spreading globally with increasing health and economic impacts.
- Many countries are still unprepared to address the challenge these diseases present and lack guidance on how and when to deploy available options for vector control intervention.
- In the absence of easy, rapid, effective solutions, the optimal approach is to intensify surveillance and to strengthen the evidence base for which tools can be best integrated for effective and proactive deployment, together with adequate support and implementation.
- IAM aims to offer practical vector control guidance on measures to prevent *Aedes*-transmitted viral diseases tailored to local entomological and epidemiological settings and available resources.
- IAM aims to promote and support implementation of the WHO GVCR and to offer guidance on improving measures to combat the emerging threat of *Aedes*-borne diseases with positive impacts on health, the economy, and the global environment.

**Top Five Papers**

- Gubler DJ. Dengue, Urbanization and Globalization: The Unholy Trinity of the 21st Century. Trop Med Health. 2011; 39 (4): 3–11.
- Achee NL, Gould F, Perkins TA, Reiner Jr RC, Morrison AC, Ritchie SA, Scott TW. A critical assessment of vector control for dengue prevention. PLoS Negl Trop Dis. 2015; 9(5): e0003655.
- World Health Organization. Comprehensive guidance for prevention and control of dengue and dengue haemorrhagic fever. Revised and expanded edition. 2011. World Health Organization, Regional Office for South-East Asia. 212 pp.
- Bowman LR, Donegan S, McCall PJ. Is dengue vector control deficient in effectiveness or evidence? Systematic review and meta-analysis. PLoS Negl Trop Dis. 2016; 10(3): e0004551.
- Runge‐Ranzinger S, McCall PJ, Kroeger A, Horstick O. Dengue disease surveillance: an updated systematic literature review. Trop Med Int Health. 2014; 19 (9):1116–60.

## Supporting information

S1 Table Entomological methods and indices used for *Aedes* surveillance. (DOCX)

S2 Table Strength of the evidence supporting vector control. (DOCX)

S3 Table Supporting activities of integrated *Aedes* management. (DOCX)
