# Correction: Integrated *Aedes* management for the control of *Aedes*-borne diseases

**DOI:** 10.1371/journal.pntd.0010310

**Published:** 2022-03-22

**Authors:** David Roiz, Anne L. Wilson, Thomas W. Scott, Dina M. Fonseca, Frédéric Jourdain, Pie Müller, Raman Velayudhan, Vincent Corbel

Fig 1 shows an incorrect length for “Risk assessment”. The authors have provided a corrected version of Fig 1 here.

**Fig 1:**
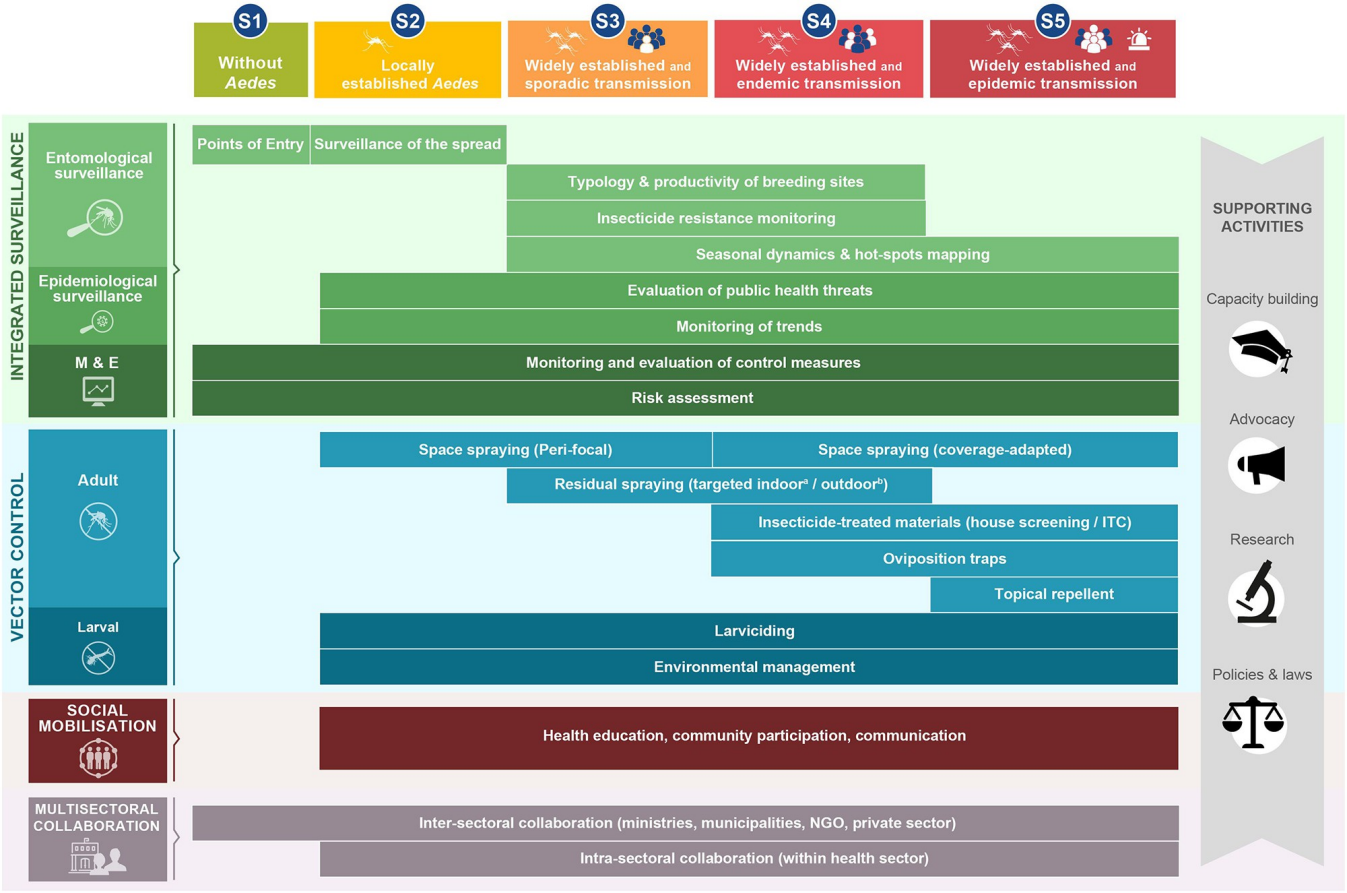
Conceptual framework of the IAM system. IAM builds on 4 pillars of activities (integrated surveillance, vector control, social mobilisation, and multisectoral collaboration) and 4 supporting activities (capacity building, advocacy, policies and laws, and research). Activities are tailored to local scenarios of *Aedes* distribution and virus transmission risk (see Box 1 for definition of terms). ^a^*A*. *aegypti;* ^b^*A*. *albopictus*. IAM, Integrated *Aedes* Management; ITC, insecticide treated curtain; M&E, monitoring and evaluation; NGO, nongovernmental organisation; S, scenario.
