# Supplementary material for: Integrated Aedes management for the control of Aedes-borne diseases
Source: PLoS Negl Trop Dis. 2018 Dec 6;12(12):e0006845. doi: 10.1371/journal.pntd.0006845 (PMC6283470; doi:10.1371/journal.pntd.0006845)
Supplement: S1 Table — (DOCX) [file pntd.0006845.s001.docx]

| **Trap methodology** | **Aim/task** | **Index name** | **Index description** | **Formula** | **Unit** | **Target** | **Strengths/weaknesses** | **References** |
| --- | --- | --- | --- | --- | --- | --- | --- | --- |
| **Ovitraps** | Surveillance of the spread & seasonal dynamics of *Aedes*.  Tailored to scenarios 1 and 2 and to a lesser extent 3 | Ovitrap index (OI) | Average proportion of positive ovitraps | Positive ovitraps / No. of ovitraps examined in a given area per month/week/ fortnight | % | Eggs | Sensitive and economical method for detecting *Aedes* introduction and/or presence in large area (surveillance).  Information not reliable for measuring *Aedes* density. | 1, 2 |
|  |  | Trap positivity index (TPI) | Proportion of positive traps | (Total no. of traps infested with eggs / Total traps) x 100 | % | Eggs |  |  |
|  |  | Egg density index (EDI) | Ratio of no. of eggs/traps | Total no. of eggs / Total no. of traps | No. of eggs per trap | Eggs | Information not reliable for measuring *Aedes* density |  |
| **Larval indices** | To measure larval abundance and characterise the typology of larval development habitats.  Tailored to scenarios 3 & 4, and to a lesser extent 2 | House index (HI), (also called premise index) | Proportion of houses positive for immature *Aedes* | (No. of houses infested / Total households) x 100 | % | Pupae, larvae | Not reliable for measuring *Aedes* population level  No information on the number of positive containers  Does not take productivity into account  Poor indication of adult production | 1, 3, 4 |
|  |  | Container index (CI) | Proportion of containers positive for immature *Aedes* | (No. of containers infested / Total containers inspected) x 100 | % | Pupae, larvae | Relevant for focussing larval control efforts and for orienting educational messages  Can provide data on larval development habitat characteristics  Does not take productivity into account  Poor indication of adult production |  |
|  |  | Breteau index (BI) | No. of *Aedes-*positive containers per 100 houses | (No. of containers infested / Total houses inspected) x 100 | No. per 100 houses | Pupae, larvae |  |  |
|  |  | Stegomya index (SI) | Proportion of positive containers per population | No of positive containers per population) x1000 | No. per 1000 people | Pupae, larvae | Not reliable for measuring *Aedes* population level, only a proxy of seasonal trends  Does not take productivity into account  Poor indication of adult production |  |
| **Pupal surveys** | To characterise the productivity of larval development habitats.  Tailored to scenarios 3 & 4 | Pupae per person index (PPI) | No. of pupae per person | No. of pupae per household population | No. per person | Pupae | Useful indicator for planning source reduction and environmental management  More relevant indicator (compared to larval indices) for estimating adult abundance and evaluating vector control interventions  Labour intensive | 1, 4, 5, 6, 7 |
|  |  | Pupa index (PI) | No. of pupae per house | No. of pupae / Total number of households inspected | % | Pupae | Same as above.  Applicable to both public and private domains |  |
|  |  | Pupae per hectare index (PHI) | No. of pupae per hectare | No. of pupae per household area | No. per hectare | Pupae |  |  |
| **Adult surveys (BG-sentinel, other traps, human landing rates)** | To measure adult abundance and monitor and evaluate vector control.  Tailored to scenarios 3,4 & 5 and to a lesser extent 1 (Points of entry) | Adult trap index (ATI) | Average no. of adults per trap and per period | Total no. of females caught / Total no. of traps | No. per trap | Adults  (indoors and outdoors) | Relevant for estimating relative abundance, seasonal dynamics and spatial distribution trends, and for evaluating vector control measures.  Labour intensive and requires skilled staff  More costly than other methods | 1, 8, 9 |
| **Sticky trap surveys** | To detect recently-introduced *Aedes* species and monitor spread.  Scenarios 1 and 2, and to a lesser extent 3,4 & 5 | Sticky trap index (STI) | No. of adults caught by the sticky trap per unit of time | (No of traps positive for *Aedes* sp./ Total no of inspected traps) x 100 | % | Adults | Poor proxy of adult abundance. Useful for collecting gravid females. Easy to use, affordable, and can be deployed at large scale.  Can be used to screen virus infections in mosquitoes | 10, 11, 12 |
| **Mosquito exposure** | Relevant for estimating vector-human contact.  For all scenarios | Mosquito exposure index (MEI) | Proportion of IgG responders to *Aedes* salivary antigen (34 kDa salivary peptide) | No. of IgG immune responders / Total number of people tested | Relative optical density (ΔOD) | Adult-human | Labour intensive and costly, requires skilled staff and laboratory capacity (ELISA)  Samples taken from dried blood spots or serum  Lack of calibration with entomological and epidemiological endpoints | 13 |
| **Adult surveys for viral detection** | Collection by BG-traps (or other methods) and subsequent RT-PCR.  Scenarios 3, 4 & 5 | Vector infection index (VII) | Proportion of Infected females | (No. of virus-infected females / Total no. of females inspected)*100 | % | Adults | Relevant for identifying the role of local species in virus transmission and/or for characterising viral strain  Costly, labour intensive, requires skilled staff and strong diagnostic capacity (RT-PCR). | 1 |
| **Adult surveys using human hosts** | Collection to estimate mosquito biting densities, adapted for monitoring control measures (taking into account ethical issues).  For scenarios 3,4,5 and to a lesser extent 2 | Human-baited double net (HDN)  or mosquito electrocuting traps (MET) | Mean number of mosquito females per person per unit of time | No. of females collected per person per unit of time | No. mosquitoes collected per person per  unit of time | Adults | Alternative techniques to the human landing catch (HLC) method for collecting anthropophagic mosquito species, and for measuring mosquito biting densities and mosquito behaviour.  Labour-intensive, costly, and may pose logistic difficulties (e.g., access to power sources)  Human-baited double traps are a good alternative to collecting outdoor mosquitoes. Mosquito electrocuting traps are a good exposure-free alternative to HLC.  HDN and MET are well accepted and pose fewer ethical problems than HLC. | 14, 15 |

References:

1. European Centre for Disease Prevention Control (ECDC). Guidelines for the surveillance of invasive mosquitoes in Europe. Technical Report. *Stockholm*: ECDC, 2012. Available from: http://ecdc.europa.eu/en/publications/Publications/TER-Mosquito-surveillance-guidelines.pdf
2. Flacio E, Engeler L, Tonolla M, Lüthy P, Patocchi N. Strategies of a thirteen year surveillance programme on *Aedes albopictus* (Stegomyia albopicta) in southern Switzerland. Parasit Vectors. 2015; 8(1): 1.

Bowman LR, Runge-Ranzinger S, McCall PJ. Assessing the relationship between vector indices and dengue transmission: a systematic review of the evidence. PLoS Negl Trop Dis. 2014; 8 (5): e2848.

1. Focks D. A review of entomological sampling methods and indicators for dengue vectors. Special Programme for Research and training in Tropical Diseases (TDR). 2003.
2. Carrieri M, Angelini P, Venturelli, C., Maccagnani, B, Bellini R. *Aedes albopictus* (Diptera: Culicidae) population size survey in the 2007 Chikungunya outbreak area in Italy. I. Characterization of breeding sites and evaluation of sampling methodologies. J Med Entomol. 2011; 48(6): 1214-1225.
3. Morrison AC, Gray K, Getis A, Astete H, Sihuincha M, Focks D, et al. Temporal and geographic patterns of *Aedes aegypti* (Diptera: Culicidae) production in Iquitos, Peru. J Med entomol. 2004; 41 (6): 1123-1142.
4. Romero-Vivas CM, Falconar A. KInvestigation of relationships between *Aedes aegypti* egg, larvae, pupae, and adult density indices where their main breeding sites were located indoors. J Am Mosq Control Assoc. 2005; 21(1): 15-21.
5. Roiz D, Duperier S, Roussel M, Boussès P, Fontenille D, Simard F, Paupy C. Trapping the Tiger: efficacy of the novel BG-Sentinel 2 with several attractants and carbon dioxide for collecting *Aedes albopictus* (Diptera: Culicidae) in Southern France. J Med Entomol. 2015; 53(2): 460-465.
6. Silver JB. Mosquito ecology: field sampling methods. Springer Science & Business Media. 2007.
7. Facchinelli L, Valerio L, Pombi M, Reiter P, Costantini C, Della Torre A. Development of a novel sticky trap for container‐breeding mosquitoes and evaluation of its sampling properties to monitor urban populations of *Aedes albopictus.* Med Vet entomol. 2007; 21(2): 183-195.
8. Gama RA, Silva EM, Silva IM, Resende MC, Eiras ÁE. Evaluation of the sticky MosquiTRAP™ for detecting *Aedes* (Stegomyia) *aegypti* (L.)(Diptera: Culicidae) during the dry season in Belo Horizonte, Minas Gerais, Brazil. Neotrop Entomology, 2007; 36(2): 294-302.
9. Ritchie SA, Long S, Hart A, Webb CE, Russell RC. An adulticidal sticky ovitrap for sampling container-breeding mosquitoes. J Am Mosq Control Assoc. 2003; 19(3): 235-242.
10. Ndille EE, Doucoure S, Poinsignon A, Mouchet F, Cornelie S, D’Ortenzio E et al. Human IgG Antibody Response to *Aedes* Nterm-34kDa Salivary Peptide, an Epidemiological Tool to Assess Vector Control in Chikungunya and Dengue Transmission Area. PLoS Negl Trop Dis. 2016; 10 (12): e0005109.
11. Tangena JA, Thammavong P, Hiscox A, Lindsay SW, Brey PT, et al. The human-baited double net trap: an alternative to human landing catches for collecting outdoor biting mosquitoes in Lao PDR. PloS One. 2015; 10 (9): e0138735.
12. Govella NJ, Maliti DF, Mlwale AT, Masallu JP, Mirzai N, Johnson PC, Killeen, GF. An improved mosquito electrocuting trap that safely reproduces epidemiologically relevant metrics of mosquito human-feeding behaviours as determined by human landing catch. Malaria J. 2016; 15(1): 465
