# Supplementary material for: Integrated Aedes management for the control of Aedes-borne diseases
Source: PLoS Negl Trop Dis. 2018 Dec 6;12(12):e0006845. doi: 10.1371/journal.pntd.0006845 (PMC6283470; doi:10.1371/journal.pntd.0006845)
Supplement: S2 Table — (DOCX) [file pntd.0006845.s002.docx]

| **Activity** | **Method** | **Level of evidence 3b:**  **Entomological impact**  **- Observational trials**  **no randomization** | **Level of evidence 3a:**  **Entomological impact- RCTs** | **Level of evidence 2b: epidemiological impact** **Observational trials** | **Level of evidence 2a: epidemiological impact** - **Controlled trials, no randomization** | **Level of evidence 1: epidemiological impact - RCTs** |
| --- | --- | --- | --- | --- | --- | --- |
| **Insecticide spraying** | **Space spraying (indoor/outdoor)** | Meta-analysis showed that outdoor space spraying reduced the BI by 75% (5 studies) but results were highly heterogeneous with a RE=0.24 (95% c.i: 0.05-1.19) (Erlanger et al., 2008).  Peridomestic space spraying showed only a short-term effect in reducing *Aedes* abundance (13 out of 15 studies) (Esu et al., 2010).  Indoor space spraying showed effective to reduce *Ae. aegypti* density but there was no lasting effect (3 studies) (Samuel et al., 2017).  Outdoor ULV fogging reduced adult density of *Ae. albopictus* in USA with a MD= -13.90 (95% c.i: -21.86, -5.94) (Bowman et al., 2016). | Indoor space spraying in combination with educational campaign produced a weak impact on *Ae. aegypti* density in Mexico (Horstick et al., 2018). | * | None | None |
|  | **Residual spraying (indoor/outdoor)** | Indoor residual spraying significantly reduced *Ae.* *aegypti* density (2 studies) (Samuel et al., 2017).  ** | None | Indoor residual spraying did not reduce dengue incidence in Taiwan and Australia (Bowman et al., 2016). | Targeted indoor residual spraying (TIRS) in potential exposure locations reduced the probability of future DENV transmission by 86-96% (Samuel et al., 2017). | None |
| **Personal protection** | **Topical repellents** | None | None | No evidence for mosquito repellents to reduce dengue incidence (1 study) (Bowman et al., 2016) | None | None |
|  | **Insecticide-treated materials (clothes, curtains, house screens, water container covers, etc.)** | None | Insecticide-Treated Curtains did not significantly reduce larval indices in a meta-analysis of 2 CRCTs (Bowman et al., 2016).  Insecticide-water tank covers significantly reduced the number of tanks positive for immature stages of *Ae. aegypti* in Sri Lanka with a MD=-4.00 (95% c.i: -4.96, -3.04) (Bowman et al. 2016).  Mixed results for Insecticide-Treated Materials (alone or in combination with chemical or biological control) on larval indices (5 studies) (Horstick et al., 2018).  Meta-analysis of studies based on Insecticide-treated window and door screens or curtains or ITND bednets did not show significant effect on BI et CI (4 studies) (Alvarado-Castro et al., 2017). | The presence of house screening in home reduced dengue incidence with an OR=0.22 (3 studies) (Bowman et al., 2016).  No evidence that mosquito bednets reduce dengue incidence (2 studies) (Bowman et al., 2016). | None | None |
| **Mass trapping** | **Oviposition traps (AGO)** | Evidence of a significant reduction on *Aedes* density using mass trapping with CDC Autocidal Gravid Ovitraps in Puerto Rico (Barrera et al., 2014; 2017). | None | Evidence of a reduction on human seropositivity and mosquito infection rate for chikungunya in treated areas (Barrera et al., 2016; Lorenzi et al., 2016). | None | None |
| **Larviciding** | **Organophosphate(Temephos)** | Evidence of a reduction in larval abundance with Temephos interventions alone (11 studies). Non conclusive results of the effectiveness of Temephos in combination with other measures, as chemical method or education campaigns (George et al., 2015). | A CRCT in Brazil showed no effect of Temephos on entomological indices (Alvarado-Castro et al., 2017). | None | None | None |
|  | **IGRs (Pyriproxyfen)** | Pyriproxyfen showed effective for reducing larval density (5 studies out of 8) (Maoz et al., 2017).  Some results suggest that auto-dissemination could be effective especially to reach cryptic breeding sites (6 studies) (Maoz et al., 2017). | One out of 2 RCTs showed significant reduction in *Ae. aegypti* larval density in Colombia (Maoz et al., 2017). | Pyriproxyfen as part of a community-based strategy reduced the rate of dengue incidence in Colombia with a RR=0.19 (95% c.i.: 0.12-0.30) (Bowman et al., 2016; Maoz et al., 2017). | None | None |
|  | **Bacillus thuriengiensis (Bti)** | Twelve studies reported reductions in entomological indices with an average duration of control of 2–4 weeks (Boyce et al., 2013). | 2 RCTs did not report significant reductions of *Aedes* densities (Boyce et al., 2013). | None | None | None |
|  | **Others (Spinosad, monomolecular film)** | None | None | None | None | None |
| **Biological control** | **General** | Meta-analysis showed that biological control (copepods, fish and *Toxorynchites*) can reduce the CI of *Aedes* with a random RE=0.18 (95% c.i: 0.07-0.44, 9 studies) (Erlanger et al., 2008). | None | None | None | None |
|  | **Fish** | There are limited evidences that the use of larvivorous fish can reduce larval density (11 studies). Combined interventions reported reduction in entomological indices (3 studies) (Han et al., 2015). | None | None | None | None |
|  | **Copepods** | 5 out of 11 community effectiveness studies (all carried out in Vietnam) showed that copepods can reduce larval and adult densities of *Ae. aegypti,* but the 6 other studies did not confirm that trend (Lazaro et al., 2015). | None | Community-based control trial in Vietnam showed that dengue incidence was reduced by 76% during the first year in treated communes (Lazaro et al. 2015). | None | None |
| **Integrated vector management with community mobilization**  **and environmental management** | **Without and with chemical or biological control** | Environmental management alone (14 studies) (removal of unused water vessels and covering of water containers) effectively reduced BI with a random RE=0.71 (95% c.i. 0.55-0.90, 9 studies), the CI with a random RE=0.43 (c.i. 0.31-0.59, 10 studies) and the HI with a random RE=0.49 (95% c.i. 0.30-0.79, 10 studies) (Erlanger et al., 2008).  Community-based strategies are able to reduce larval density (11 studies) (Heintze et al., 2007).  Meta-analysis showed that Environmental management coupled with chemical control effectively reduced larval indexes with a random RE=0.33 (95% c.i. 0.22-0.48, 11 studies) for the BI and with a random RE= 0.12 (95% c.i. 0.02-0.62, 8 studies) for the HI (Erlanger et al., 2008).  Clean-up with IRS and larviciding reduced ovitrap positivity in Australia with a MD = -10.30 (95% c.i: -12.80, -7.80) (Bowman et al., 2016).  Fogging, source reduction and larviciding reduced BI (OR = 0.15,95% c.i:  0.10, 0.24) and HI (OR = 0.13, 95% c.i: 0.08, 0.22) in Argentina (Bowman et al., 2016). | The impacts of community mobilization (CRCTs) were broadly positive with a RE of -0.1 (95% c.i. -0.2; 0.00, 4 studies) for HI, -0.03 (95% c.i. -0.05;-0.01) (3 studies) on CI, -0.13 (95% c.i. -0.22;-0.05, 4 studies) on BI (Alvarado-Castro et al., 2017).  3 CRCTs reported significant reductions of *Aedes* densities with Community-based interventions (Bowman et al., 2016). | Community based environmental management together with the use of water containers covers reduced dengue risk with a OR=0.22 (95% c.i., 0.15–0.32, p<0.0001) (Bowman et al., 2016). | None | Community mobilization + clean-up lower risk of dengue infection in children with a RR reduction of 29.5% (95% c.i. 3.8- 55.3%) and self-reports of dengue of 24.7% (95% c.i. 1.8-51.2%). *** (Alvarado-Castro et al., 2017). |
| **Community mobilization** | **Educational messages embedded in a community-based approach** | Educational messages embedded in a community-based approach reduced larval indexes. Regression analysis (22 studies) leads to a RE=0.25 (95% c.i. 0.17-0.37) (Al Muhandis et al., 2011).  Combined community participation coupled with other dengue control tools (larvicides, fish, copepods) can reduce larval indices (5 studies) (Heintze et al. 2007). | None | Dengue incidence was reduced for community participation coupled with copepods in Vietnam and coupled with chemical larvicides in Thailand (Heintze et al. 2007). | None | None |

**Footnote:** We used scores to rank the “strength of evidence” based on study designs used for assessing the efficacy of vector control interventions as proposed by Wilson et al (2015) for epidemiological trials (1, 2a, 2b). We created 2 new “levels of evidence” (3a and 3b) to distinguish randomized versus non-randomized (observational) “entomology” trials. As presented in the Table, details of the studies (as quantification of evidence, number of studies, existence of pooled analysis or location was specified). **IGR**: Insect Growth Regulator. **BI:** Breteau Index. **HI**: House Index. **CI**: Container Index. **OR:** odds ratio. **MD:** Median difference. **RR**: Relative Risk. **RE:** Relative Effectiveness. **c.i:** confidence interval. By means of a meta-analysis, Erlanger et al (2008) estimated the effectiveness of different dengue vector control interventions, defined as the relative reduction of an entomological measure caused by the intervention compared with the control (no intervention) or the pre-intervention phase (baseline situation before intervention). Additional studies not included in the different reviews: *Stoddard et al., 2014 *in* Bowman et al. (2016): a longitudinal study analyzing twelve years of data from the city of Iquitos in Peru which concluded that dengue cases could be reduced if intensive city-wide space-spraying (outdoor fogging) was conducted early in the transmission season. Samuel et al. (2017) also commented this study but it was excluded in both cases. **Outdoor residual spraying reduced *Ae. albopictus* populations in Australia but this study was not included in the reviews (Muzari et al., 2017). ***These results were exposed in Andersson et al., 2015 in Alvarado-Castro et al., 2017. Meta-analysis are specified otherwise no pooled analyses were calculated.

**References:**

Al-Muhandis N, Hunter PR. The value of educational messages embedded in a community-based approach to combat dengue fever: a systematic review and meta regression analysis. PLoS Negl Trop Dis. 2011; 5 (8): e1278.

Alvarado-Castro V, Paredes-Solís S, Nava-Aguilera E, Morales-Pérez A, Alarcón-Morales L, Balderas-Vargas NA, et al. Assessing the effects of interventions for Aedes aegypti control: systematic review and meta-analysis of cluster randomised controlled trials. BMC Public Health. 2017 May 30;17(Suppl 1):384. doi: 10.1186/s12889-017-4290-z.

Andersson N, Nava-Aguilera E, Arosteguí J, Morales-Perez A, Suazo-Laguna H, Legorreta-Soberanis J et al. Evidence based community mobilisation for dengue prevention in Nicaragua and Mexico (Camino Verde, the Green Way): cluster randomized controlled trial. BMJ. 2015; 351: h3267.

Barrera R, Acevedo V, Felix GE, Hemme RR, Vazquez J, Munoz JL, Amador M. Impact of Autocidal Gravid Ovitraps on Chikungunya Virus Incidence in *Aedes aegypti* (Diptera: Culicidae) in Areas With and Without Traps. J Med Entomol. 2017; 54 (2): 387-395.

Barrera R, Amador M, Acevedo V, Caban B, Felix G, Mackay AJ. Use of the CDC autocidal gravid ovitrap to control and prevent outbreaks of Aedes aegypti (Diptera: Culicidae). J Med Entomol. 2014 Jan;51(1):145-54.

Bowman LR, Donegan S, McCall PJ. Is dengue vector control deficient in effectiveness or evidence? Systematic review and meta-analysis. PLoS Negl Trop Dis. 2016; 10 (3): e0004551.

Boyce R, Lenhart A, Kroeger A, Velayudhan R, Roberts B, Horstick O. Bacillus thuringiensis israelensis (Bti) for the control of dengue vectors: systematic literature review. Trop Med Int Health. 2013 May;18(5):564-77. doi: 10.1111/tmi.12087.

Erlanger TE, Keiser J, Utzinger J. Effect of dengue vector control interventions on entomological parameters in developing countries: a systematic review and meta‐analysis. Med Vet Entomol. 2008; 22 (3): 203-221.

Esu E, Lenhart A, Smith L, Horstick O. Effectiveness of peridomestic space spraying with insecticide on dengue transmission; systematic review. Trop Med Int Health. 2010; 15 (5), 619-631.

George L, Lenhart A, Toledo J, Lazaro A, Han WW, Velayudhan R, et al. Community-Effectiveness of Temephos for Dengue Vector Control: A Systematic Literature Review. PLoS Negl Trop Dis. 2015 Sep 15;9(9):e0004006. doi: 10.1371/journal.pntd.0004006

Han WW, Lazaro A, McCall PJ, George L, Runge-Ranzinger S, Toledo J, et al. Efficacy and community effectiveness of larvivorous fish for dengue vector control. Trop Med Int Health. 2015 Sep;20(9):1239-1256. doi: 10.1111/tmi.12538. Epub 2015 Jun 3.

Heintze C, Garrido MV, Kroeger A. What do community-based dengue control programmes achieve? A systematic review of published evaluations. T Roy Soc Trop Med H. 2007; 101 (4): 317-325.

Horstick O, Runge-Ranzinger S. Protection of the house against Chagas disease, dengue, leishmaniasis, and lymphatic filariasis: a systematic review. Lancet Infect Dis. 2018 May;18(5):e147-e158. doi: 10.1016/S1473-3099(17)30422-X.

Lazaro A, Han WW, Manrique-Saide P, George L, Velayudhan R, Toledo J, et al. Community effectiveness of copepods for dengue vector control: systematic review. Trop Med Int Health. 2015 Jun;20(6):685-706. doi: 10.1111/tmi.12485

Lorenzi OD, Major C, Acevedo V, Perez-Padilla J, Rivera A, Biggerstaff BJ, Munoz-Jordan J, Waterman S, Barrera R, Sharp TM. Reduced Incidence of Chikungunya Virus Infection in Communities with Ongoing *Aedes Aegypti* Mosquito Trap Intervention Studies - Salinas and Guayama, Puerto Rico, November 2015-February 2016. MMWR Morb Mortal Wkly Rep. 2016 May 13;65(18):479-80. doi: 10.15585/mmwr.mm6518e3.

Maoz D, Ward T, Samuel M, Müller P, Runge-Ranzinger S, Toledo J, et al. Community effectiveness of pyriproxyfen as a dengue vector control method: A systematic review. PLoS Negl Trop Dis. 2017 Jul 17;11(7):e0005651. doi: 10.1371/journal.pntd.0005651

Muzari MO, Devine G, Davis J, Crunkhorn B, van den Hurk A, Whelan P, et al. Holding back the tiger: Successful control program protects Australia from Aedes albopictus expansion. PLoS Negl Trop Dis. 2017 Feb 13;11(2):e0005286. doi: 10.1371/journal.pntd.0005286.

Samuel M, Maoz D, Manrique P, Ward T, Runge-Ranzinger S, Toledo J. Community effectiveness of indoor spraying as a dengue vector control method: A systematic review. PLoS Negl Trop Dis. 2017; 11(8).

Stoddard ST, Wearing HJ, Reiner Jr RC, Morrison AC, Astete H, Vilcarromero S et al. Long-term and seasonal dynamics of dengue in Iquitos, Peru. PLoS Negl Trop Dis. 2014; 8: e3003.
