# Supplementary material for: Integrated Aedes management for the control of Aedes-borne diseases
Source: PLoS Negl Trop Dis. 2018 Dec 6;12(12):e0006845. doi: 10.1371/journal.pntd.0006845 (PMC6283470; doi:10.1371/journal.pntd.0006845)
Supplement: S3 Table — (DOCX) [file pntd.0006845.s003.docx]

| **Supporting activity** | **Description** | **Specifications / recommendations** | **References** |
| --- | --- | --- | --- |
| Capacity-building | Aims to increase capacity and capability in vector control and surveillance, systematic insecticide resistance monitoring, etc. Staff must be carefully selected and trained, given adequate remuneration, clearly-defined roles and responsibilities, and opportunities for career progression. Many countries should strengthen their capacity for pesticide registration to ensure sound management of public health insecticides. | The strategy must be developed long before an outbreak occurs. Logistics, trained vector control professionals, funds and administrations with strong leadership are needed to ensure interventions are effective. Countries should follow WHO guidelines for testing and evaluating PH insecticides and monitoring insecticide resistance, and should endorse the WHO-FAO Code of Conduct on Pesticide Management. The WHO Tropical Disease Research Programme (TDR) can build the capacity of national partners, support individual career development and strengthen institutions in the field of disease and vector surveillance and control. | 1, 2, 3, 4, 5, 6 |
| Policies and laws | These refer to any changes in judicial/legal systems to enforce sanitary legislation and regulations to prevent the introduction and/or proliferation of mosquitoes. This includes enforcing mosquito control legislation in support of control strategies, (e.g., door-to-door visits and environmental management), making changes to local or regional legislation and, in some cases, issuing penalties for encouraging the breeding of mosquitoes. International legislation, such as the International Health Regulations (WHO, 2008), has been drawn up to guide countries on preventing the introduction and spread of diseases and their vectors through regulatory functions and requirements for mosquito control and surveillance at international ports, airports and border crossings. | Legislation can contribute to limiting *Aedes* development and spread by targeting specific larval development habitats, such as tyres. Regulations can prohibit constructions or equipment likely to facilitate proliferation of mosquitoes (gutters, roof terraces, etc.). It can also require specific equipment, such as mosquito-proof devices on water tanks, cisterns and septic tanks. Authorities can decide to subsidise personal protection devices (by reducing the costs of coils and repellents or the taxes on them) during epidemics. Legislation can mandate reporting of cases of *Aedes*-borne diseases by health care personnel. | 2, 7, 8, 9 |
| Research | Basic and applied research should be encouraged to improve vector control. Research should focus on vector biology and ecology and other population parameters, such as host preferences, vector dispersal and longevity, resting habitats, and vector competence and insecticide resistance. National research centres should be involved in gathering evidence, and data should be analysed and disseminated to competent authorities to guide decision making. | Basic research is needed to better characterise local *Aedes* populations and develop locally-adapted vector control tools. Applied research takes many forms including, for example, testing/evaluating new vector control tools. Trials of these tools should be conducted rigorously and should include epidemiological outcomes, given that there is to date only weak evidence regarding *Aedes* vector control. Innovation should be encouraged in developing new surveillance tools and strategies, such as using remotely-sensed climate data, human movement or internet data to predict outbreaks. Applied research should also focus on methods for improving community education and mobilisation, and on strategies for integrating the non-health sector in *Aedes* control. Implication of MoH can move the research agenda towards applied and important research topics. | 2, 3, 10, 11, 12, 13, 14 |
| Advocacy | Advocacy is a process through which groups of stakeholders can be influenced to gain their support for and reduce barriers to specific initiatives or programmes. Advocacy is important in making the case for *Aedes* control. The case should also be made for the benefits of inter-sectoral collaboration, community involvement, effectiveness and cost effectiveness of vector control, and potential cost savings. Multiple strategies, often deployed simultaneously, are the key to the success of any advocacy effort and efforts should target national and international stakeholders. | Administrative advocacy relies on effective communication flow among stakeholders and decision-makers from local, regional, national and international organisations. Developing educational material on vector control and personal protection measures is recommended. Media advocacy must be taken into account in the communication plan. It is essential to keep the message simple and to communicate the key actions to be undertaken. | 2, 8, 15 |

**References:**

1. World Health Organization. Global Vector Control Response 2017-2030. 53 pp. Geneva: World Health Organization. 2017. Available at : <http://www.who.int/vector-control/publications/global-control-response/en/>

2. World Health Organization. Comprehensive guidance for prevention and control of dengue and dengue haemorrhagic fever. Revised and expanded edition. World health Organization, Regional Office for South-East Asia. 212 pps. 2011. Available from: http://apps.searo.who.int/pds_docs/B4751.pdf

3. European Centre for Disease Prevention Control (ECDC). Guidelines for the surveillance of invasive mosquitoes in Europe. Technical Report. *Stockholm*: ECDC, 2012. Available from: http://ecdc.europa.eu/en/publications/Publications/TER-Mosquito-surveillance-guidelines.pdf

4. World Health Organization. Handbook for integrated vector management. World Health Organization. 2012. Available from: http://apps.who.int/iris/bitstream/10665/44768/1/9789241502801_eng.pdf

5. Escadafal C, Gaayeb L, Riccardo F, Pérez-Ramírez E, Picard M, Dente MG, et al. Risk of Zika virus transmission in the Euro-Mediterranean area and the added value of building preparedness to arboviral threats from a One Health perspective. BMC Public Health. 2016; 16 (1):1219.

6. Spiegel SJ, Veiga MM. Building capacity in small-scale mining communities: health, ecosystem sustainability, and the Global Mercury Project. EcoHealth, 2005; 2(4): 361-369.

7. Horstick O, Runge-Ranzinger S, Nathan MB, Kroeger A. Dengue vector-control services: how do they work? A systematic literature review and country case studies. T Roy Soc Trop Med H. 2010; 104 (6): 379-386.

8. Heintze C, Garrido MV, Kroeger A. What do community-based dengue control programmes achieve? A systematic review of published evaluations. T Roy Soc Trop Med H. 2007; 101 (4): 317-325.

9. Camargo S. History of *Aedes aegypti* eradication in the Americas. Bull World Health Organ. 1967; 36: 602-603.

10. Bowman LR, Donegan S, McCall PJ. Is dengue vector control deficient in effectiveness or evidence? Systematic review and meta-analysis. PLoS Negl Trop Dis. 2016; 10 (3): e0004551.

11. Achee NL, Gould F, Perkins TA, Reiner Jr, RC, Morrison AC, Ritchie SA, Scott TW. A critical assessment of vector control for dengue prevention. PLoS Negl Trop Dis. 2015; 9 (5): e0003655.

12. Wilson AL, Boelaert M, Kleinschmidt I, Pinder M, Scott TW, Tusting LS. Lindsay SW. Evidence-based vector control? Improving the quality of vector control trials. Trends Parasitol. 2015; 31(8): 380-390.

13. Bonizzoni M, Gasperi G, Chen X, James AA. The invasive mosquito species *Aedes albopictus*: current knowledge and future perspectives. Trends Parasitol. 2013; 29 (9): 460-468.

14. Paupy C, Delatte H, Bagny L, Corbel V, Fontenille D. *Aedes albopictus,* an arbovirus vector: from the darkness to the light. Microbes Infect. 2009; 11(14): 1177-85.

15. Parks W, Lloyd LS. Planning social mobilisation and communication for dengue fever prevention and control: a step-by-step guide. Geneva, World Health Organization, 2004 (available from: <http://www.who.int/tdr/publications/publications/pdf/planning_dengue.pdf>).
